# Urinary Aromatic Amino Acid Metabolites Associated With Postoperative Emergence Agitation in Paediatric Patients After General Anaesthesia: Urine Metabolomics Study

**DOI:** 10.3389/fphar.2022.932776

**Published:** 2022-07-19

**Authors:** Yueyue Li, Jingjie Li, Yuhuan Shi, Xuhui Zhou, Wanqing Feng, Lu Han, Daqing Ma, Hong Jiang, Yongfang Yuan

**Affiliations:** ^1^ Department of Pharmacy, Shanghai Ninth People’s Hospital, Shanghai JiaoTong University School of Medicine, Shanghai, China; ^2^ Department of Anaesthesiology, Shanghai Ninth People’s Hospital, Shanghai Jiao Tong University School of Medicine, Shanghai, China; ^3^ Division of Anaesthetics, Pain Medicine and Intensive Care, Department of Surgery and Cancer, Faculty of Medicine, Imperial College London, Chelsea and Westminster Hospital, London, United Kingdom

**Keywords:** aromatic amino acids, emergence agitation, metabolomics, paediatric anaesthesia, prediction model, urine neurotransmitters

## Abstract

**Background:** Emergence agitation (EA) is very common in paediatric patients during recovery from general anaesthesia, but underlying mechanisms remain unknown. This prospective study was designed to profile preoperative urine metabolites and identify potential biomarkers that can predict the occurrence of EA.

**Methods:** A total of 224 patients were screened for recruitment; of those, preoperative morning urine samples from 33 paediatric patients with EA and 33 non-EA gender- and age-matched patients after being given sevoflurane general anaesthesia were analysed by ultra-high-performance liquid chromatography (UHPLC) coupled with a Q Exactive Plus mass spectrometer. Univariate analysis and orthogonal projection to latent structures squares-discriminant analysis (OPLS-DA) were used to analyse these metabolites. The least absolute shrinkage and selection operator (LASSO) regression was used to identify predictive variables. The predictive model was evaluated through the receiver operating characteristic (ROC) analysis and then further assessed with 10-fold cross-validation.

**Results:** Seventy-seven patients completed the study, of which 33 (42.9%) patients developed EA. EA and non-EA patients had many differences in preoperative urine metabolic profiling. Sixteen metabolites including nine aromatic amino acid metabolites, acylcarnitines, pyridoxamine, porphobilinogen, 7-methylxanthine, and 5′-methylthioadenosine were found associated with an increased risk of EA, and they all exhibited higher levels in the EA group than in the non-EA group. The main metabolic pathways involved in these metabolic changes included phenylalanine, tyrosine and tryptophan metabolisms. Among these potential biomarkers, L-tyrosine had the best predictive value with an odds ratio (OR) (95% CI) of 5.27 (2.20–12.63) and the AUC value of 0.81 (0.70–0.91) and was robust with internal 10-fold cross-validation.

**Conclusion:** Urinary aromatic amino acid metabolites are closely associated with EA in paediatric patients, and further validation with larger cohorts and mechanistic studies is needed.

**Clinical Trial Registration:** clinicaltrials.gov, identifier NCT04807998

## 1 Introduction

Emergence agitation (EA), also known as emergence delirium, is common in paediatric patients during recovery from general anaesthesia. EA is characterized by crying, screaming, agitation, disorientation, and altered responses to their environment (Koch et al., 2018). EA paediatric patients may cause themselves physical injury and stress to care workers and their patients and prolong postoperative recovery time. Furthermore, young patients who had EA after general anaesthesia and surgery were reported to have more likely behavioural changes that may last for several weeks (Lee and Sung, 2020).

The mechanisms of paediatric EA remain unclear. Several risk factors including age, preoperative anxiety, anaesthesia type and postoperative pain were reported (Voepel-Lewis et al., 2003). Indeed, sevoflurane, an inhaled anaesthetic commonly used in paediatric surgery, was found to be closely related to the incidence of EA (Cohen et al., 2003; Chandler et al., 2013). Sevoflurane stimulated locus coeruleus neurons, increased lactic acid and glucose concentrations in the parietal cortex and induced asymptomatic epileptogenic activity (Vlajkovic and Sindjelic, 2007; Gibert et al., 2012; Jacob et al., 2012; Stoicea et al., 2014), all of which may trigger EA, but these are assumptions and further study is needed.

Metabolomics is an emerging analytical system biology methodology that studies the metabolic changes of endogenous metabolites in living systems under pathological conditions or in response to medications (Carneiro et al., 2019). Metabolomics has been commonly used to explore biomarkers in paediatric neurological diseases such as epilepsy, autism, attention deficit and hyperactivity disorder (Kennedy et al., 2019; Zhou et al., 2019; Wang L. J. et al., 2021). Metabolomics was also applied to preclinical studies of anaesthetic-induced neurotoxicity in paediatric patients, which was considered to be an effective approach to identify biomarkers of adverse events associated with anaesthesia (Liu et al., 2015; Wang et al., 2018). Wang Q et al. (2021) used the metabolomics method to explore the preoperative serum biomarkers of emergence agitation after general anaesthesia in adult patients and found that choline, cytidine, glycophosphorophocholine, L-phenylalanine, oleamide and inosine may have a predictive value for EA diagnosis. In addition, metabolomic techniques have also been applied to the discovery of predictive biomarkers for postoperative delirium in elderly patients. Abnormal changes in the levels of multiple metabolites in the preoperative serum or cerebrospinal fluid in elderly patients were found to be associated with the occurrence of postoperative delirium (Watne et al., 2016; Guo et al., 2017; Han et al., 2020). Postoperative emergence agitation is the postoperative delirium phenotype in the young population. Up to date, it remains unknown whether metabolic changes in young patients are associated with EA after anaesthesia and surgery. The aim of this study, therefore, was to determine metabolites associated with an increased risk of EA in paediatric patients through preoperative urine metabolomic profiling analyses.

## 2 Methods

### 2.1 Chemicals and Reagents

HPLC-grade acetonitrile and formic acid were purchased from Tedia (Fairfield, OH, United States). 3-Hydroxykynurenine and 3-hydroxyanthranilic acid were obtained from Sigma-Aldrich (St. Louis, MO). Tyrosine and xanthurenic acid were purchased from Shanghai Aladdin Bio-Chem Technology Co., LTD.

### 2.2 Participants

This prospective observational cohort study protocol (SH9H-2020-T149) was approved by the Institutional Review Board of the Ninth People’s Hospital of Shanghai. The study was part II of the prospective study entitled “Risk factors for paediatric emergence agitation and postoperative pain following maxillofacial surgery and analysis of serum or urine metabolomics in children with agitation—an exploratory study” and was registered at ClinicalTrials.gov (NCT04807998). The study was conducted at the Departments of Anaesthesiology, Otolaryngology, and Oral and Maxillofacial Surgery of the Ninth People’s Hospital of Shanghai Jiaotong University’s School of Medicine from April 2021 to September 2021. It was in compliance with the Declaration of Helsinki for Medical Research. Written informed consent was obtained from parents or guardians of all patients before recruitment.

Paediatric patients aged 3–7 years with an American Society of Anaesthesiologists (ASA) physical status of I or II who were scheduled for tonsillectomy and/or adenoidectomy under sevoflurane-based anaesthesia were recruited. The exclusion criteria included the presence of 1) developmental delays or intellectual disability; 2) a history of neurological or psychiatric diseases; 3) severe liver and kidney dysfunction or other heart and lung diseases; 4) metabolic diseases or inherited diseases; 5) surgery with total intravenous anaesthesia or combined intravenous-inhalation anaesthesia and; 6) under medications or healthy supplements, e.g. vitamins.

### 2.3 Anaesthesia and Assessment of EA

All patients had fasted from 22:00 onward before surgery. They all received routine premedications of dolasetron 0.3–0.6 mg/kg and dexamethasone 0.25–0.3 mg/kg (i.v. injection). Non-invasive blood pressure, ECG and pulse oximetry were continuously monitored throughout anaesthesia and surgery. Anaesthetic induction included the sequential administration of intravenous midazolam, propofol, fentanyl and rocuronium to facilitate endotracheal intubation. Anaesthesia maintenance was 1.5%–2.5% sevoflurane supplemented with an intravenous administration of remifentanil (0.05–0.3 μg/kgmin) when necessary to maintain a bispectral index range from 40 to 60. Patients were transferred to the post-anaesthetic care unit (PACU) after surgery completion. The tracheal tube was removed in PACU when spontaneous breathing and swallowing cough reflexes were fully recovered.

During recovery, emergence agitation and postoperative pain were assessed and recorded according to the Paediatric Assessment of Emergence Delirium (PAED) score and the Face, Legs, Activity, Cry and Consolability scale (FLACC) by a researcher who was blinded with the study protocol. PAED and FLACC scores were assessed every 10 min until the patient returned to the ward. Emergence agitation was considered once the patient had a PAED score equal to or greater than 12. Once EA occurred, they were treated with esketamine 0.1–0.25 mg/kg, sufentanil 0.05–0.1 μg/kg or fentanyl 0.5–1 μg/kg administered intravenously as necessary.

### 2.4 Data Collection

**FIGURE 1:**
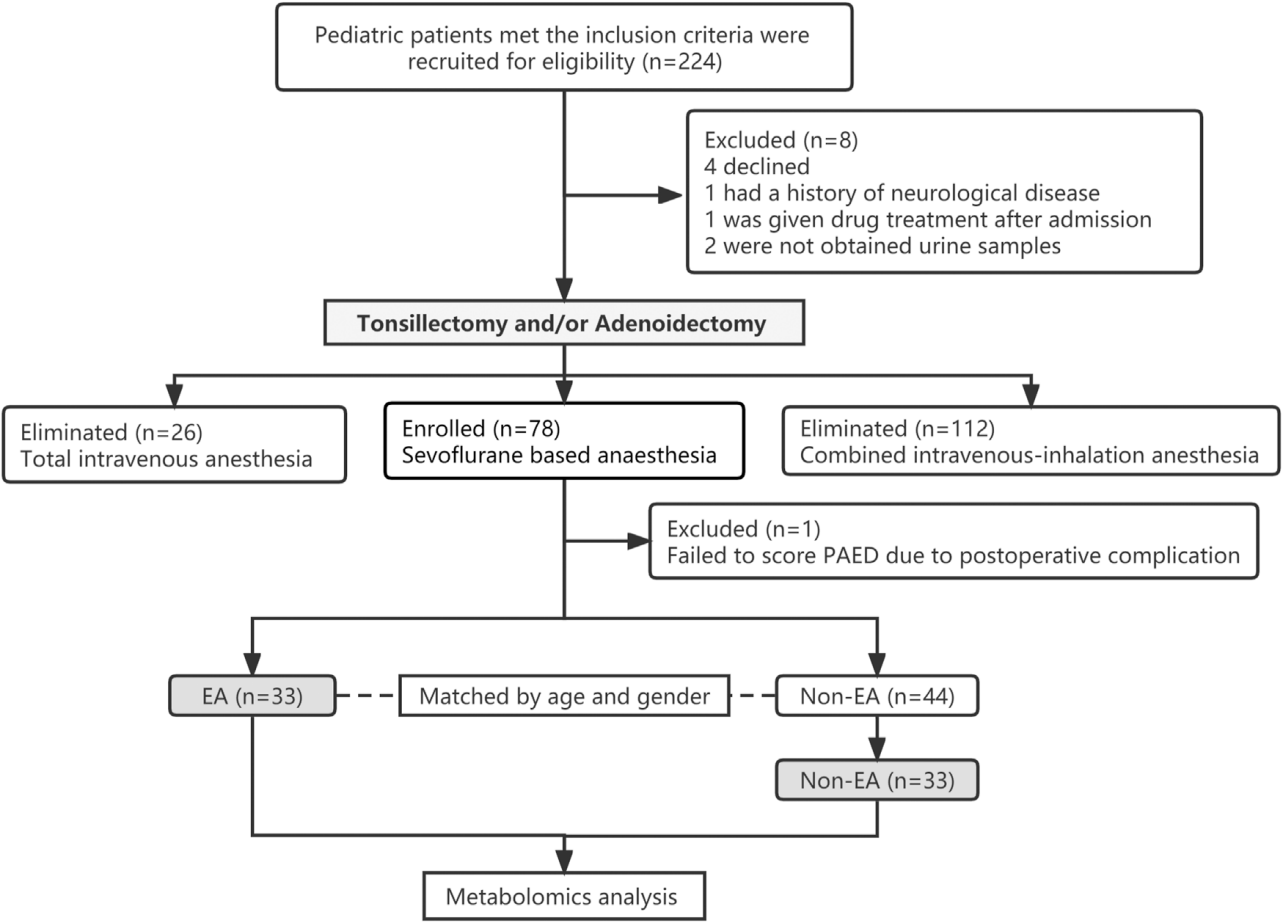
CONSORT flow diagram of the study.

Once EA patients were identified, gender- and age-matching non-EA patients, who had the same type of surgery in a similar time period, were randomly selected to match the same number of EA patients for metabolomics analysing comparisons (Figure 1). Patients’ demographics in both groups including age, gender, height, weight, BMI, ASA physical status classification, length of surgery, anaesthesia or anaesthetic dose were collected.

### 2.5 Study End Points

The primary outcome of our study was the usefulness of preoperative urine in metabolomics in predicting the occurrence of postoperative EA. The secondary outcomes were FLACC scores and post-operative complications or adverse events including postoperative nausea and vomiting (PONV), respiratory (e.g., cough, laryngospasm and bronchospasm) and cardiovascular (bradycardia and hypotension) complications.

### 2.6 Urine Sample Collection and Metabolomic Profiling

A first morning urine sample from each patient was collected on the operation day. Urine samples were centrifuged at 13,000 g at 4 C for 10 min, and then, the supernatants were kept at −80°C for further analyses. Prior to liquid chromatography–mass spectrometry (LC–MS) analysis, 400 μL acetonitrile was added to 100 μL aliquots of freshly thawed urine samples. The mixture was, then, vortexed for 2 min and centrifuged at 13,000 g for 10 min at 4°C. The supernatant was transferred to vials for the LC–MS analysis. To monitor the stability of instrument analysis, a quality control (QC) sample was made by combining 10 μL aliquots of each urine sample. Before analysing the entire list of samples, the QC sample was injected six times consecutively to balance the system. Then, the QC sample was injected into every ten samples throughout the entire sample analysis. Variables with a coefficient of variation (CV) ≥ 20% in QC samples were excluded from the data matrix.

The metabolite analysis was performed using a Vanquish UHPLC system (Thermo Fisher) coupled with a Q Exactive Plus mass spectrometer (Thermo Fisher, Milan, Italy) (Lavarello et al., 2021). An InfinityLab Poroshell 120 EC-C18 (2.1 × 100 mm, 2.7 μm, Agilent) column was used for chromatographic separation. The column temperature was set at 45°C. Ultrapure water with 0.1% formic acid (v/v) and acetonitrile (Tedia, Fairfield, OH, United States) with 0.1% formic acid (v/v) constituted phase A and phase B of the mobile phase, respectively. The gradient elution programme for urine analysis was as follows: 1% B (0–1 min), 1%–20% B (1–5.5 min), 20%–30% B (5.5–6 min), 30%–35% B (6–8.5 min), 35%–70% B (8.5–10.5 min), 70%–100% B (10.5–11 min), 100% B (11–13.5 min) and 1% B (13.5–15.5 min). The flow rate was 0.4 ml/min, and the sample injection volume was 1 μL.

Mass detection was performed using a heated electrospray source probe both in positive and negative modes with the following settings: scan mode: DDA mode, one full scan followed by five MS/MS scans. The scan range was set from 67 to 1,000 m/z. The scanning resolutions of MS1 and MS2 were 70,000 (at m/z 200) and 17,500, automatic gain control was 1 × 10^6^ and 5 × 10^5^, and the maximum injection times were 100 ms and 50 ms, respectively. The spray voltages in positive and negative modes were 3,200 and 2800 V, respectively. The auxiliary gas temperature was 300°C, and the capillary temperature was 320°C. Sheath gas and auxiliary gas were set at 30 and 10 a.u., respectively. The s-lens RF level was 50 V. The collision energy was set to 15 and 30 to fragment the ions.

Samples were analysed in a random manner. All the data were acquired by Xcalibur 3.0 (Thermo Fisher). Then, the LC–MS raw data were converted *via* Progenesis QI v2.3 software (Waters Corp., Milford, MA, United States) for peak finding and alignment. Compound identification was achieved by comparing the precision m/z, MS2, RT and other information collected by mass spectrometry with authentic standards or searching the Human Metabolome Database (http://hmdb.ca/) or METLIN (https://metlin.scripps.edu/).

### 2.7 Study Power

Due to the complexity of untargeted metabolomics, there are currently no standard methods for sample size estimation. Given that this was a prospective preliminary study, we randomly recruited and collected samples with 33 patients per group, which met the requirement for adequate statistical modelling of metabolomics study (Nyamundanda et al., 2013).

### 2.8 Statistical Analysis

#### 2.8.1 Clinical Data

Continuous variables such as age, weight, BMI, duration of anaesthesia and surgery, and anaesthetic dose were reported as the mean ± SD. Categorical variables including demographic variables were reported as frequencies and percentages. PAED scores were reported as median (interquartile range; IQR). Data were analysed with the chi-squared test, Student t-tests or Mann–Whitney *U* test with SPSS 23.0 software (SPSS, Inc.) where appropriate. A *p*-value less than 0.05 was considered to be of statistical significance.

#### 2.8.2 Metabolomics Data

The urine creatinine level of each sample was measured and used to normalize the urinary metabolomics data. Any metabolites that were not detected in more than 50% urine samples were excluded from further analysis (Hackstadt and Hess, 2009). Then, metabolites for which data had zero values (due to below of the threshold of MS detection, for example) were imputed with the minimum detection level of that metabolite (Dehaven et al., 2010). The univariate analysis by independent Student’s t test was applied to analyse differences of variables between EA and non-EA groups with SPSS 23.0 software (SPSS, Inc.). The *p*-values were corrected with the false discovery rate (FDR) calculated with the “fdrtool” package in R 4.1.2. The principal component analysis (PCA) and the orthogonal projection to latent structures squares-discriminant analysis (OPLS-DA) were performed with the data matrix *via* SIMCA-P 14.1 (Umetrics, Umea, Sweden) software. PCA was used to obtain an overview of metabolic profiling. OPLS-DA was conducted to identify differential biomarkers between EA and non-EA groups. R2Y (the interpretation rate parameter), Q2 (the predictive ability parameter) and a random permutation test were used to indicate the quality of the OPLS-DA model. The variables with variable importance for projection (VIP) scores >1 in the OPLS-DA model, a *p*-value <0.05 (FDR <5%) for independent sample *t* test and the FC value (the ratio of mean values of EA and non-EA groups) > 1.5 were selected as differential metabolites. Metabolic pathways of differential metabolites were analysed by MetaboAnalyst 4.0 and KEGG.

#### 2.8.3 Development and Validation of Predictive Models

Variable selection was performed with the differential metabolites obtained through univariate analysis and OPLS-DA modelling. The levels of metabolites were log-transformed (base 10) and mean-centred and divided by the standard deviation of each variable. The least absolute shrinkage and selection operator (LASSO) regression using a 10-fold cross-validation was used to select the most predictive metabolites to EA (Zhu, et al., 2022). The selection was judged from the lambda with 1 SE of the minimum partial likelihood of deviance. The LASSO regression method was performed in R with the “glmnet” package. The performance of the metabolite biomarker model was evaluated through the receiver operating characteristic (ROC) analysis and underwent internal validation using the 10-fold cross-validation. The area under the ROC curve (AUC), sensitivity and specificity values, as well as predictive values were used to assess the predictive power of potential metabolite biomarker panels.

## 3 Results

### 3.1 Demographics and Clinical Characteristics of EA and Non-EA Groups

A total of 224 paediatric patients were enrolled in the study; however, 147 patients were excluded for the following reasons: 4 declined, 1 had a history of neurological disease, 1 was given drug treatment after admission, 2 were not obtained urine samples, 26 with total intravenous anaesthesia, 112 with combined intravenous-inhalation anaesthesia and 1 failed to score PAED due to post-operative complication. Ultimately, 77 patients completed the study, and their morning urine samples were obtained (Figure 1). Among them, 33 (42.9%) patients developed EA in PACU. Another 33 age- and gender-matched patients without EA were selected to comprise the non-EA group. There were no significant differences in age, gender, height, weight, BMI, length of surgery, anaesthesia or anaesthetic dose between patients in EA and non-EA groups (Table 1). PAED scores and FLACC scores in the EA group were significantly higher than those of the non-EA group (*p* < 0.01). The incidence of post-operative adverse reactions that occurred in PACU such as PONV, cough, laryngospasm and bronchospasm was not statistically different between the two groups (*p* > 0.05).

**TABLE 1:** Characteristics of post-operative emergence agitation (EA) and non-EA patients.

Characteristic	EA group (*n* = 33)	Non-EA group (*n* = 33)	*p*-value
Age(y)	4.45 ± 1.12	4.97 ± 1.29	0.088
Male, n (%)	22 (66.7%)	21 (63.6%)	0.796
Height, cm	111.21 ± 8.12	115.06 ± 11.74	0.126
Weight, kg	20.84 ± 5.50	22.36 ± 7.09	0.333
BMI	16.59 ± 2.24	16.53 ± 2.45	0.913
Surgical time, min	26.85 ± 15.87	25.73 ± 14.11	0.763
Anaesthesia duration, min	37.70 ± 18.13	35.36 ± 16.40	0.585
Midazolam premedication (mg kg^−1^)	0.051 ± 0.011	0.048 ± 0.014	0.426
Rocuronium bromide usage (mg kg^−1^)	0.78 ± 0.16	0.79 ± 0.12	0.972
Propofol induction (mg kg^−1^)	2.30 ± 0.37	2.23 ± 0.29	0.370
Fentanyl induction (μg kg^−1^)	1.95 ± 0.24	2.06 ± 0.24	0.067
Remifentanil maintenance (μg kg^−1^min^−1^)	0.097 ± 0.082	0.073 ± 0.086	0.258
Bolus of fentanyl dosage (μg kg^−1^)	1.38 ± 1.05	1.30 ± 0.56	0.727
MAC concentration	0.62 ± 0.32	0.62 ± 0.15	0.942
PAED score^a^	17 (18–15)	4 (7–3)	<0.001^c^
FLACC score^b^	2 (4–0.5)	0 (0–0)	<0.001^d^
Case of PONV	2 (6.1%)	1 (3.0%)	1.000
Case of cough/laryngospasm/bronchospasm	5 (15.2%)	0 (0.0%)	0.063

BMI: body mass index; MAC: minimum alveolar concentration; PAED: Paediatric Assessment of Emergence Delirium; FLACC: Face, Legs, Activity, Cry and Consolability scale; PONV: postoperative nausea and vomiting.

a PAED and FLACC scores were reported as median (interquartile range).

b PAED and FLACC scores were reported as median (interquartile range).

c Mann–Whitney *U* test was used to compare PAED and FLACC scores between EA and non-EA groups.

d Mann–Whitney *U* test was used to compare PAED and FLACC scores between EA and non-EA, groups.

### 3.2 Metabolic Profiles of EA and Non-EA Groups

**FIGURE 2:**
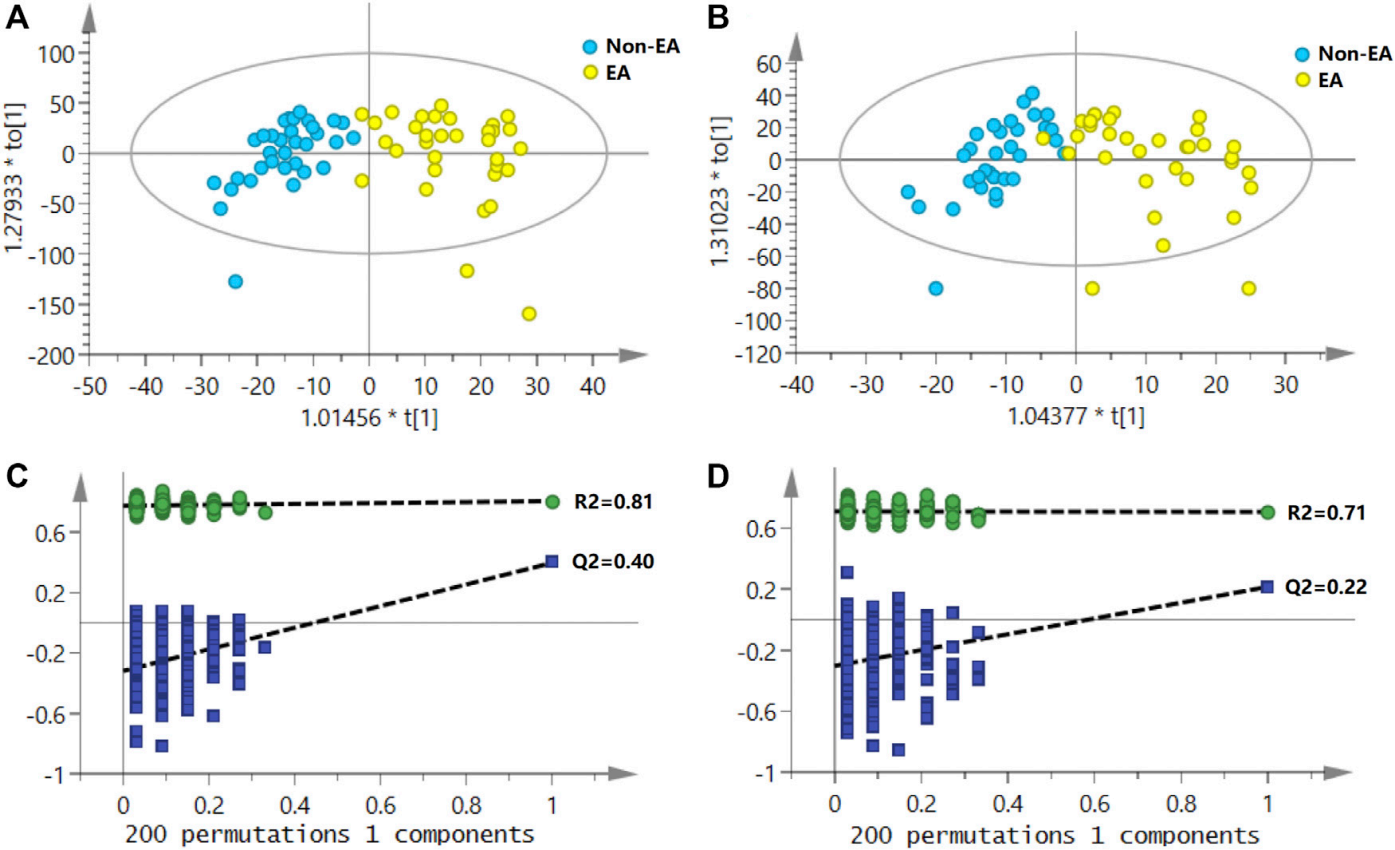
Score plots of OPLS-DA for the urine metabolomics of emergence agitation (EA) versus the non-EA patients; **(A)** the positive ionization mode; **(B)** the negative ionization mode. The permutation test (200 tests total) for the OPLS-DA model; **(C)** in the positive ionization mode, R2Y and Q2Y were 0.81 and 0.40, respectively; **(D)** in the negative ionization mode, R2Y and Q2Y were 0.71 and 0.22, respectively.

A typical urine LC–MS total ion chromatogram is shown in Supplementary Figure S1. As shown in PCA score plots (Supplementary Figure S2), QC samples were clustered closely together, indicating the high stability of the LC–MS system during the entire sequence. As shown in Figure 2, the urine samples of the EA group and the non-EA group were clearly divided into two clusters in the OPLS-DA score plots. In the positive ion mode, OPLS-DA model parameters R2Y and Q2 were 0.81 and 0.40, respectively. In the negative ion mode, OPLS-DA model parameters R2Y and Q2 were 0.71 and 0.22, respectively. The corresponding 200-fold randomization permutation test results showed that OPLS-DA models were valid without overfitting.

### 3.3 Metabolomic Analysis and Metabolite Predictive Models for EA

**FIGURE 3:**
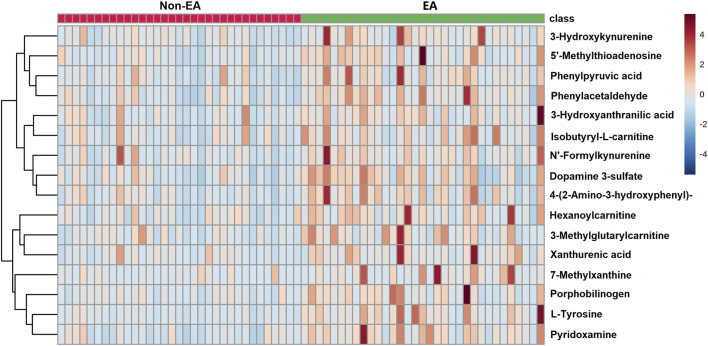
Heat map of differential metabolites between emergence agitation (EA) and non-EA patients.

In our study, metabolites with VIP >1, *p* < 0.05 (FDR<5%) and FC > 1.5 were selected as potential biomarkers. As listed in Table 2, 16 metabolites associated with an increased risk of EA were identified, of which nine metabolites were aromatic amino acids and their metabolites. Others included several acylcarnitine, pyridoxamine, porphobilinogen, 7-methylxanthine and 5′-methylthioadenosine. The heatmap of differential metabolites between EA and non-EA groups is shown in Figure 3. All the 16 metabolites exhibited higher levels in the EA group than in the non-EA group. Using the metabolic pathway analysis function of MetaboAnalyst 5.0, several related metabolic pathways with *p* < 0.05 were found, including phenylalanine metabolism, phenylalanine, tyrosine and tryptophan biosynthesis, and tryptophan metabolism.

**TABLE 2:** Potential biomarkers of urine samples from postoperative emergence agitation (EA) and non-EA patients.

Cluster	Metabolites	RT (min)	Measured m/z	VIP^a^	FC^b^	*p*-value^c^	FDR*^d^*	Pathway
Amino acid and metabolites	L-Tyrosine	1.53	199.1073	2.47	2.52	<0.001	0.004	Tyrosine metabolism
	Phenylacetaldehyde	1.23	121.0645	2.10	1.62	<0.001	0.006	Phenylalanine metabolism
	Dopamine 3-sulphate	1.18	216.0321	1.95	1.56	0.001	0.013	Tyrosine metabolism
	4-(2-Amino-3-hydroxyphenyl)-2,4-dioxobutanoate	4.33	206.0443	1.92	1.71	<0.001	0.011	Tryptophan metabolism
	Phenylpyruvic acid	1.07	182.0809	1.68	1.59	0.003	0.021	Phenylalanine metabolism
	Xanthurenic acid	4.93	228.0262	1.68	1.93	0.008	0.033	Tryptophan metabolism
	3-Hydroxyanthranilic acid	3.10	154.0496	1.56	1.69	0.014	0.045	Tryptophan metabolism
	3-Hydroxykynurenine	1.01	225.0865	1.51	1.64	0.005	0.026	Tryptophan metabolism
	N′-Formylkynurenine	3.08	237.0864	1.35	1.61	0.017	0.049	Tryptophan metabolism
Vitamin	Pyridoxamine	2.97	169.0968	2.07	1.77	<0.001	0.004	Vitamin B6 metabolism
Nucleosides	5′-Methylthioadenosine	4.43	298.0960	2.05	1.69	0.001	0.013	Cysteine and methionine metabolism
Organic nitrogen	Porphobilinogen	3.49	227.1021	1.88	2.10	0.002	0.017	Porphyrin and chlorophyll metabolism
Acylcarnitine	3-Methylglutarylcarnitine	3.56	290.1590	1.70	2.02	0.006	0.027	Fatty acid metabolism
	Hexanoylcarnitine	6.31	260.1850	1.61	1.57	0.004	0.025	Fatty acid metabolism
	Isobutyryl-L-carnitine	3.67	232.1537	1.37	1.55	0.013	0.044	Fatty acid metabolism
Purine derivative	7-Methylxanthine	1.07	167.0560	1.35	3.18	0.012	0.041	Caffeine metabolism

a VIP: variable importance in the projection in the OPLS-DA model. Metabolites are sorted by their VIP value under each cluster.

b Fold change (FC) was calculated from the normalized peak area between the EA group vs. the non-EA group.

c The *p*-value was calculated from Student’s *t*-test.

d The false discovery rate (FDR) was calculated from the “fdrtool” package in the R environment.

**FIGURE 4:**
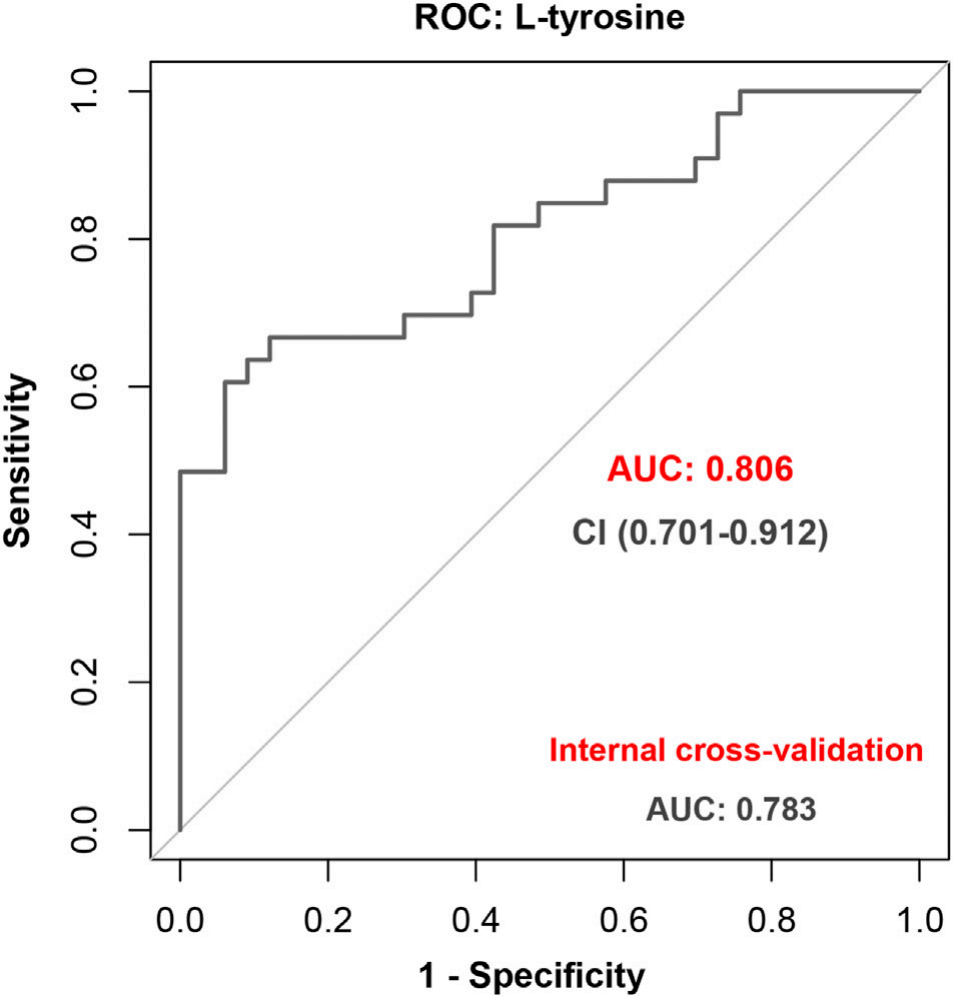
Performance of L-tyrosine to predict emergence agitation (EA) with the receiver operating characteristic (ROC) analysis and further internal cross-validation.

The LASSO regression selected the two most predictive metabolites, L-tyrosine and pyridoxamine (Supplementary Figure S3). Then, the backward stepwise logistic regression was used to examine the association between the two metabolites and occurrence of EA. The result showed that only L-tyrosine (*p* = 2.0 × 10^–4^) was retained in the model. The odds ratio (OR) (95% CI) was 5.27 (2.20–12.63). The AUC of ROC (95% CI) was 0.81 (0.70–0.91). The sensitivity and specificity were 0.67 (0.51–0.83) and 0.88 (0.77–0.99), respectively. The positive predictive value (PPV) and negative predictive value (NPV) were 0.846 and 0.725, respectively. At internal 10-fold cross-validation, the model was shown to be robust with an AUC of 0.78 (0.67–0.90) (Figure 4).

## 4 Discussion

This is the first metabolomics study that demonstrated that EA patients and non-EA patients can be distinguished by pre-operative urine metabolic profiling. We found 16 metabolites including aromatic amino acids, acylcarnitines, pyridoxamine, porphobilinogen, 7-methylxanthine and 5′-methylthioadenosine were associated with the EA incidence, implying that the occurrence of EA in paediatric patients was associated with different neuroactive metabolites and the disturbance of the overall balance of metabolic pathways. Furthermore, L-tyrosine was found to be effectively predicting EA in paediatric patients.

Of 5,005 metabolites in urine, 16 metabolites were identified to be associated with an increased risk of EA in paediatric patients; nine metabolites of those were aromatic amino acids and their metabolites. The main metabolic pathways involved in these metabolic changes included phenylalanine, tyrosine and tryptophan metabolisms. These three are all essential aromatic amino acids that are precursors of monoamine neurotransmitters such as catecholamines and serotonin. It has been reported that the neuroactive metabolite level changes, and the disturbance of the overall balance of metabolic pathways were closely related to the occurrence of neurological diseases (Fernstrom and Fernstrom, 2007; Pan et al., 2018).

In view of the limited accessibility to the central nervous system (CNS) in patients, peripheral biomarkers in blood or body fluids related to nervous system diseases were increasingly explored to indirectly reflect the disease-associated changes in the CNS. As a non-invasive and easily available body fluid, urine has been widely used for these purposes. Indeed, previous studies have indicated a relationship between urinary neurotransmitter metabolites and neurotransmitter levels in the CNS in rats (Chekhonin et al., 2000; Lynn-Bullock et al., 2004). The usefulness of urinary neurotransmitter metabolites as biomarkers of neurological diseases or disorders was documented in paediatric patients (Gevi et al., 2016; Yu et al., 2021). Sevoflurane anaesthesia was reported to be closely related to the incidence of EA (Cohen et al., 2003; Chandler et al., 2013). The underlying mechanisms of this association remain unknown, but it may relate to neurotransmitter imbalances including serotonin (5-HT) and neuropeptide Y (NPY) induced by inhalational agents such as isoflurane or sevoflurane administration reported in the pre-clinical studies (Westphalen et al., 2013; Kang et al., 2020).

The kynurenine pathway (KP) is the major metabolic pathway of tryptophan. Along KP, tryptophan is the first to be metabolised to N′-formyl-l-kynurenine, which is then further metabolised to be L-kynurenine (KYN) and 3-hydroxy-l-kynurenine (3-HK). According to our data, the levels of 3-HK and its further metabolites 3-hydroxyanthranilic acid (3-HAA), 4-(2-amino-3-hydroxyphenyl)-2,4-dioxobutanoate and xanthurenic acid in urine of the EA group were higher than those of the non-EA group. Both 3-HK and 3-HAA are considered to be neurotoxic metabolites. They inhibit the T-cell response through the formation of intracellular reactive oxygen species or glutathione depletion or activate glutamate receptors to cause headache (Lee et al., 2010; Curto et al., 2015). Xanthurenic acid can indirectly activate the mGlu2/3 metabotropic glutamate receptor and inhibit the vesicular glutamate transporter (Fazio et al., 2017). Increasing evidence indicated that the activation of tryptophan metabolites, especially those in KP, was involved in neurocognitive disorders under inflammatory conditions of the central nervous system (Watzlawik et al., 2016).

Phenylalanine is a precursor of catecholamines and indispensable for the biosynthesis of these neurotransmitters. In the present study, we noted that the amounts of phenylpyruvic acid and phenylacetaldehyde, by-products of phenylalanine, were increased in urine in the EA group patients. Phenylpyruvic acid inhibited the activity of glucose-6-phosphate dehydrogenase, hindered NADPH production and likely changed the redox status of brain cells (Rosa et al., 2012). These results indicated that dysregulated phenylalanine metabolism may likely be one of the pathogeneses of EA.

The excretion of tyrosine was also increased in the urine of the EA group in our study. The increased tyrosine level led to an increase in dopamine (DA) formation (Woods and Meyer, 1991). We found an increased production of DA metabolite dopamine 3-sulphate in the urine of the EA group, indicating that the concentration of DA was likely increased in our young patients’ blood. The increase in DA transmission accompanied by a decrease of cholinergic transmission is one of the most common neurotransmitter imbalances in the pathogenesis of delirium (Maldonado, 2013). Compared with the non-EA group, the urine excretion of aromatic amino acids and their metabolites were increased in the EA group, indicating the high monoaminergic activity of the EA group. Interestingly, in line with our data, the increase levels of aromatic amino acids in the cerebrospinal fluid were also found in elderly patients who developed delirium post-operatively (Watne et al., 2016; Han et al., 2020), whilst post-operative emergence agitation is the post-operative delirium phenotype in the young population.

In addition, several other metabolites, for example, pyridoxamine, were found to be increased in the urine of the EA group. Pyridoxamine is one of the three interconvertible members of vitamin B6. All three forms are bio-transformed into physiologically active pyridoxal-5-phosphate which has multiple functions. It is an essential coenzyme/cofactor in processes including metabolism of essential amino acids, glycogen and lipid metabolism, and synthesis of neurotransmitters such as serotonin, dopamine and GABA (Parra et al., 2018). 3-Methylglutarylcarnitine, isobutyryl-L-carnitine and hexanoylcarnitine are acylcarnitine. Acylcarnitine and carnitine supplementation had beneficial effects in the treatment of various neurological diseases. Neuroprotective benefits of brain acylcarnitines included improving mitochondrial function, increasing the antioxidant activity, stabilizing membrane composition, modulating protein and genes and enhancing cholinergic neurotransmission (Zanelli et al., 2005; Silva-Adaya et al., 2008). However, how much of these metabolites contribute to the EA development in our patients remains unknown and warrants further study.

Our study has several limitations. The number of subjects included in this study was relatively small. In addition, the patients we included in the study were mainly OSAS patients. It has been reported that urinary neurotransmitter metabolites were altered in children with OSAS and may predict cognitive morbidity (Kheirandish-Gozal et al., 2013). All these may induce the less generalizability of our findings. However, despite the aforementioned limitations, the strictly matched analysis between EA and non-EA patients was carried out, and the metabolite profile in urine reported here for predicting paediatric patients with EA may help understand the underlying mechanisms of postoperative EA for preventive strategy development in the future.

In conclusion, we found certain metabolites including aromatic amino acids, acylcarnitines, pyridoxamine, porphobilinogen, 7-methylxanthine and 5′-methylthioadenosine were closely associated with the EA incidence, and L-tyrosine was found to be effectively predicting EA in paediatric patients. The clinical significance of our work is subjected to further study in a large cohort of patients.

## Data Availability

The raw data supporting the conclusion of this article will be made available by the authors, without undue reservation.
