# Supplementary material for: Urinary Aromatic Amino Acid Metabolites Associated With Postoperative Emergence Agitation in Paediatric Patients After General Anaesthesia: Urine Metabolomics Study
Source: Front Pharmacol. 2022 Jul 19;13:932776. doi: 10.3389/fphar.2022.932776 (PMC9343964; doi:10.3389/fphar.2022.932776)
Supplement: Supplementary file 1 [file DataSheet1.docx]

Supplementary Material

#
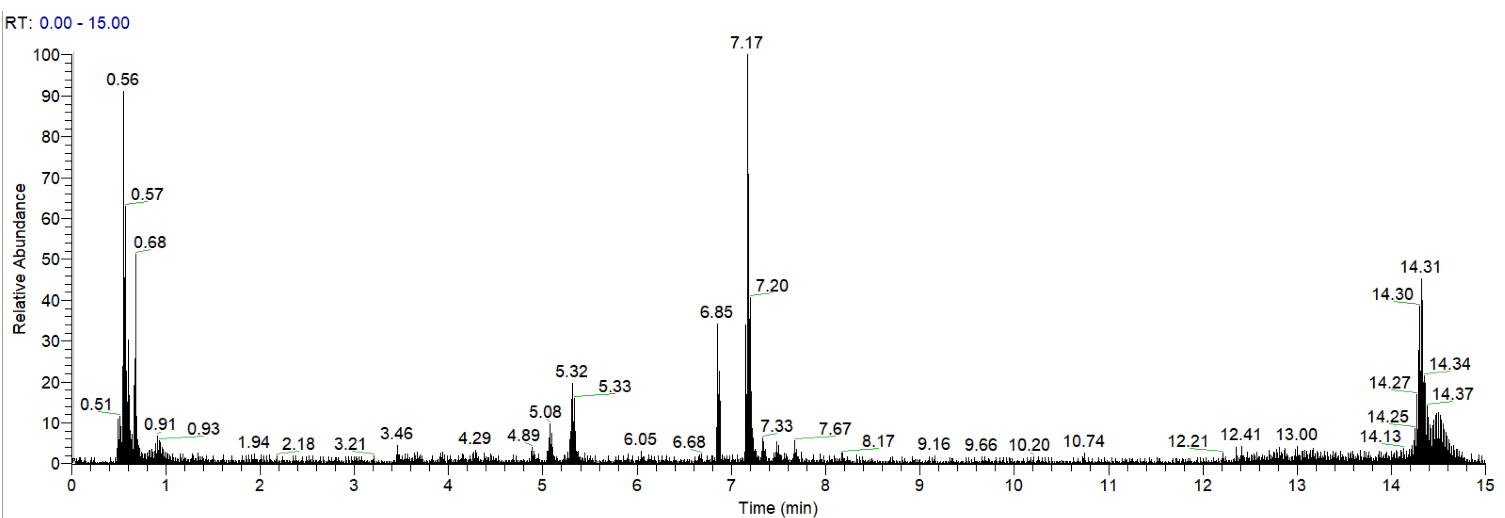
Supplementary Figures

**Supplementary Figure 1.** A typical urine LC-MS total ion chromatogram


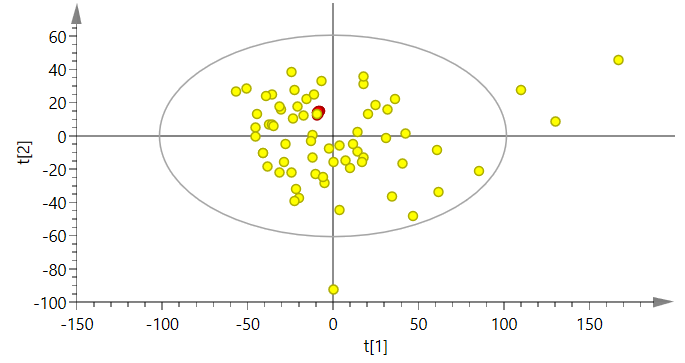


**Supplementary Figure 2.** The score plot of PCA analysis in positive ionization mode. The red clustered dots represent QC samples.


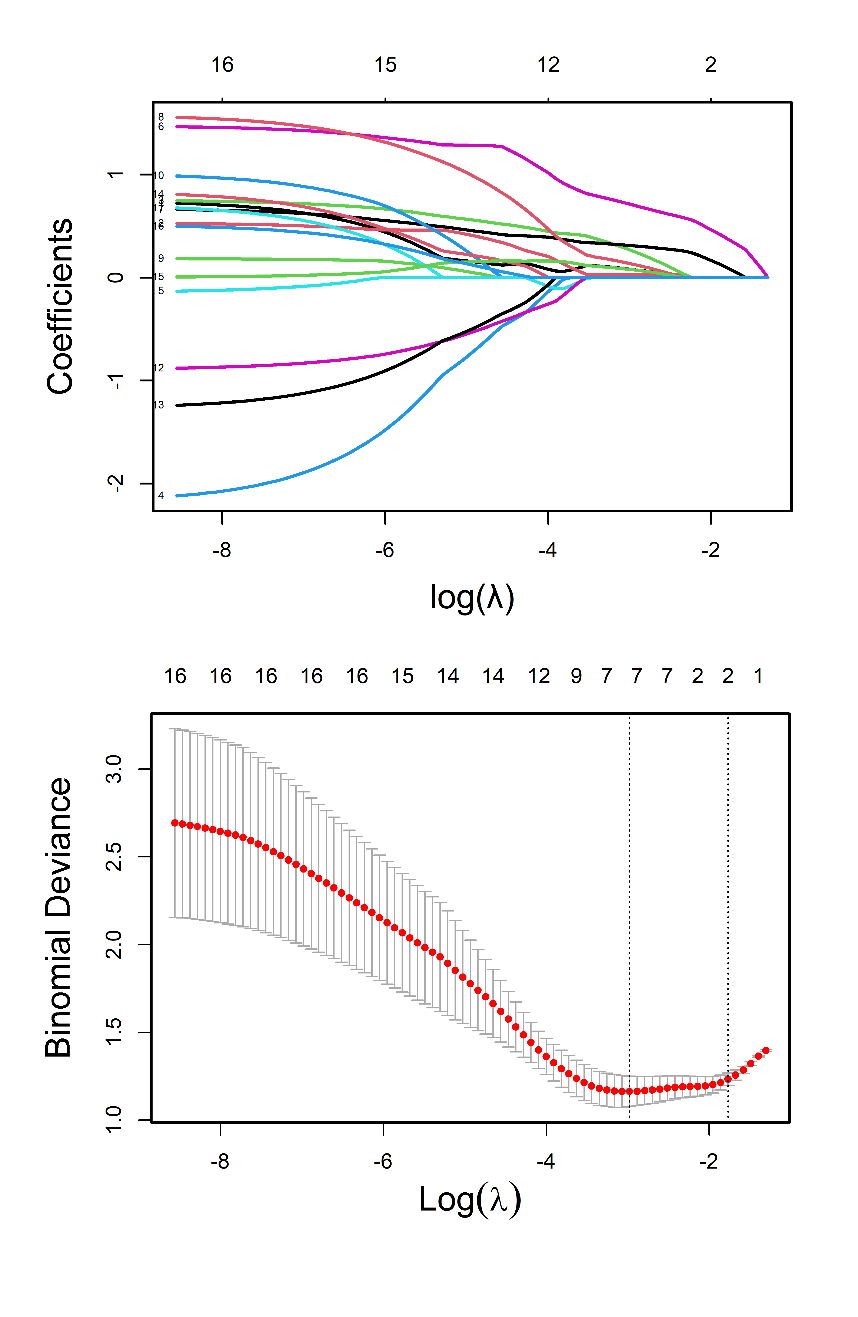
**Supplementary Figure 3.** The least absolute shrinkage and selection operator (LASSO) regression was used to select the most predictive metabolites to EA.
